# Wild Bird Densities and Landscape Variables Predict Spatial Patterns in HPAI Outbreak Risk across The Netherlands

**DOI:** 10.3390/pathogens11050549

**Published:** 2022-05-06

**Authors:** Janneke Schreuder, Henrik J. de Knegt, Francisca C. Velkers, Armin R. W. Elbers, Julia Stahl, Roy Slaterus, J. Arjan Stegeman, Willem F. de Boer

**Affiliations:** 1 Department of Population Health Sciences, Faculty of Veterinary Medicine, Utrecht University, 3584 CL Utrecht, The Netherlands; janneke.schreuder@wur.nl (J.S.); j.a.stegeman@uu.nl (J.A.S.); 2 Wildlife Ecology and Conservation Group, Wageningen University & Research, 6708 PB Wageningen, The Netherlands; henjo.deknegt@wur.nl (H.J.d.K.); fred.deboer@wur.nl (W.F.d.B.); 3 Department of Epidemiology, Bioinformatics and Animal Models, Wageningen Bioveterinary Research, 8221 RA Lelystad, The Netherlands; armin.elbers@wur.nl; 4 Sovon, Dutch Centre for Field Ornithology, 6525 ED Nijmegen, The Netherlands; julia.stahl@sovon.nl (J.S.); roy.slaterus@sovon.nl (R.S.)

**Keywords:** avian influenza, influenza A virus, highly pathogenic avian influenza, poultry, disease outbreaks, surveillance, wild-domestic interface, spatial modelling, random forest

## Abstract

Highly pathogenic avian influenza viruses’ (HPAIVs) transmission from wild birds to poultry occurs globally, threatening animal and public health. To predict the HPAI outbreak risk in relation to wild bird densities and land cover variables, we performed a case-control study of 26 HPAI outbreaks (cases) on Dutch poultry farms, each matched with four comparable controls. We trained machine learning classifiers to predict outbreak risk with predictors analyzed at different spatial scales. Of the 20 best explaining predictors, 17 consisted of densities of water-associated bird species, 2 of birds of prey, and 1 represented the surrounding landscape, i.e., agricultural cover. The spatial distribution of mallard (*Anas platyrhynchos*) contributed most to risk prediction, followed by mute swan (*Cygnus olor*), common kestrel (*Falco tinnunculus*) and brant goose (*Branta bernicla*). The model successfully distinguished cases from controls, with an area under the receiver operating characteristic curve of 0.92, indicating accurate prediction of HPAI outbreak risk despite the limited numbers of cases. Different classification algorithms led to similar predictions, demonstrating robustness of the risk maps. These analyses and risk maps facilitate insights into the role of wild bird species and support prioritization of areas for surveillance, biosecurity measures and establishments of new poultry farms to reduce HPAI outbreak risks.

## 1. Introduction

Highly pathogenic avian influenza A viruses (HPAIVs) of clade 2.3.4.4 have spread globally, causing massive outbreaks in commercial poultry farms [1,2], especially in recent years. The migratory movements of wild birds have been shown to play an important role in the global spread of HPAIVs, as spatial and temporal patterns of outbreaks coincided with migratory flyways and the timing of autumn migration [1,3]. Some HPAIV subtypes, e.g., H5N8 (in 2014 and 2016) and H5N6 (in 2017), have caused outbreaks in Europe on commercial poultry farms, as well as massive mortality in wild birds [4,5,6,7,8,9]. HPAI outbreaks were reported in more than 30 EU countries, mostly with subtypes H5N8 and H5N5, H5N1 and H5N3 in 2020–2021, and dominated by H5N1 from 2021–2022, with detections in four geographical regions, i.e., Europe, Africa, the Americas and Asia [2,10,11,12,13,14]. Most of these cases were reported in wild birds, primarily in waterbirds such as the barnacle goose (*Branta leucopsis*), graylag goose (*Anser anser*), mute swan (*Cygnus olor*), common buzzard (*Buteo buteo*) and several species of ducks (*Anatidae*) and gulls (*Laridae*) [15,16]. 

These recurrent and massive outbreaks of HPAI underline the need for better prediction of HPAI risk areas to reduce outbreak risk and take appropriate measures. HPAI outbreaks on poultry farms are spatially associated with the proximity of waterbodies or the presence of wild birds [17,18,19,20]. For example, the magnitude of the increase in densities of HPAI high risk bird species nearby HPAIV H5N8 infected poultry farms in the Dutch wetlands in the fall and winter of 2016–2017 was significantly higher compared to the non-infected farms in non-water-rich areas [20]. This was especially true for the Eurasian wigeon (*Mareca penelope*), which was one of the species with massive mortality due to HPAIV in 2016–2017 [6]. In addition, dead wild birds found at sites in the vicinity of the HPAIV infected poultry farms had phylogenetically related viruses, which may suggest that HPAIV on these farms originated from the infected wild birds [4,21]. HPAIV introduction into poultry farms most likely results from indirect contact with wild birds, and it is hypothesized that the virus enters the poultry farm via vectors or fomites contaminated with wild bird feces [21,22,23,24,25]. This suggests that wild bird presence and density can be used as predictor in identifying HPAI high risk areas.

Previously, disease distribution models showed that both land cover, particularly the presence of wetlands, and environmental variables could be used to successfully predict HPAI outbreak risk [17,18,19]. However, landscape variables are likely to be merely a proxy for the presence and density of wild birds. We hypothesize that including wild bird densities in HPAI outbreak risk modelling, rather than only landscape variables, increases the accuracy of the prediction of HPAI outbreaks. 

Several factors complicate the prediction and understanding of the spatial patterns in HPAI outbreak risk. First, associations between outbreak risk and spatial predictors may be spatial scale dependent. Second, machine learning algorithms can be used as a ‘black box’, where either the underlying patterns are not considered, or associations can even be unrealistic and non-interpretable, such as when predicted HPAI risk decreases with increasing wild bird density. Lastly, spatial predictors often show high levels of multicollinearity, for instance, the densities of many waterbird species are often positively correlated, which complicates statistical routines. 

For a water(fowl)-rich country, such as the Netherlands, it is especially challenging to determine which areas are more at risk than others. The aim of this study was to identify wild bird species and land cover variables that are associated with HPAI outbreak risk on poultry farms to study the spatial pattern of HPAI outbreak risk across the Netherlands and generate HPAI risk maps. For this purpose, we used a highly detailed dataset on wild bird densities, obtained from a structured and elaborate system of bird counts across the whole country. Furthermore, we used machine learning classifiers to predict outbreak risk at different spatial scales. In the model building process and analyses we explicitly considered the scale dependency between predictors and HPAI outbreak risk, the correlation structure between predictors, and only allowed monotonously increasing relationships between the wild bird densities and HPAI outbreak risk. 

This study contributes to a better understanding of the factors determining HPAI outbreak risk, and a better prediction of spatial patterns. This will help support prioritization of areas for surveillance, biosecurity measures and establishment of new poultry farms, to reduce HPAI outbreak risks.

## 2. Results

**Figure 1:**
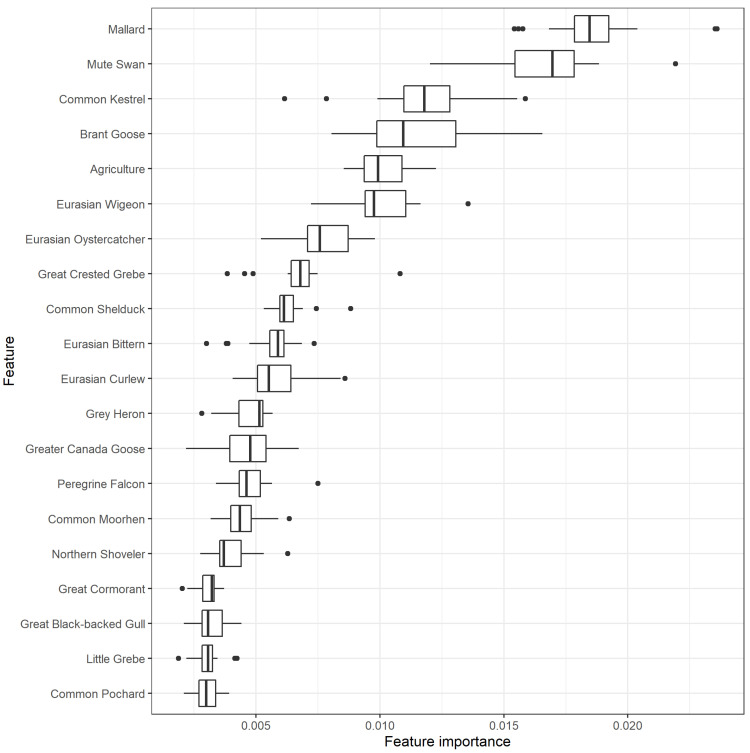
Feature importance of the 20 most important scale-aggregated predictors (Table S1) in the random forest using leave-one-group-out cross validation (LOGO-CV). The boxplots indicate the variation in feature importance across the different LOGO-CV iterations.

A total of 56 features (all 5 land cover features and 51 bird species), from the initial 5 land cover and 58 high risk HPAI bird species, passed the filtering stage. Twenty features had higher-than-average feature importance in the random forest classifier (Figure 1). All but one of these were bird densities, with agricultural cover being the only landscape variable with higher-than-average importance. All of the most important features showed the strongest univariate associations to HPAI outbreak risk at larger scales of spatial smoothing (2.5 and 5 km). 

According to the random forest classifier, the spatial distribution of mallard (*Anas platyrhynchos*) contributed most to the prediction of HPAI risk, followed by mute swan (*Cygnus olor)*, common kestrel (*Falco tinnunculus*) and brant goose (*Branta bernicla*).

**Figure 2:**
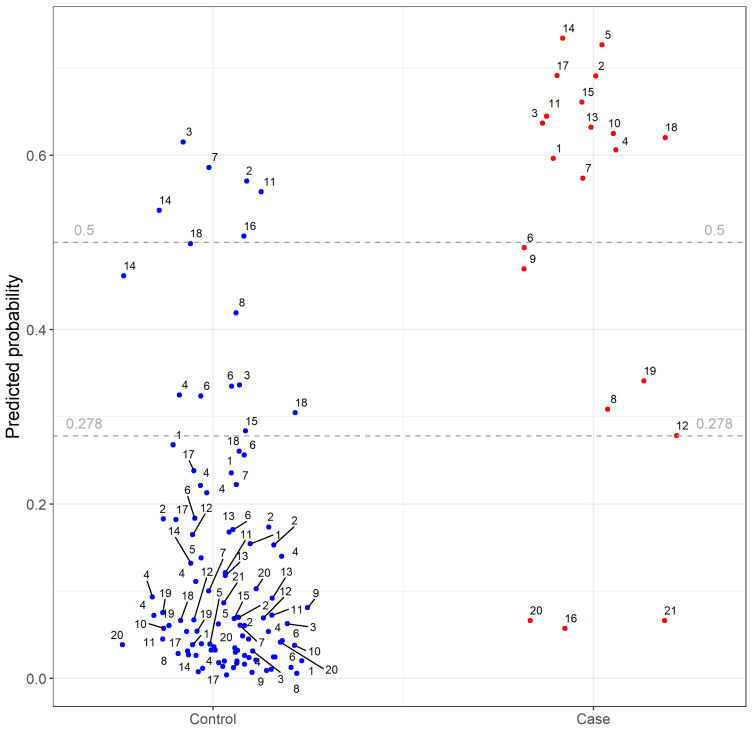
Results of final leave-one-group-out random forest (LoGo random forest). Each dot represents an individual highly pathogenic avian influenza case farm (red) or control poultry farm (blue). The number labels indicate case-ID of each farm. The predicted probability is given for each case and control farms within a set, after training of the LoGo random forest on the remaining cases and controls. The horizontal lines represent different cut-off values for test performance analyses of which 0.278 is the weighted F1 score.

The random forest with all multiscale predictors showed high predictability: an overall accuracy of 0.86, sensitivity (recall) of 0.88 and an area under the receiver operating characteristic curve (AUC-ROC) of 0.92 (Figure 2; Table 1). The gradient boosted decision tree generated even better predictions (accuracy = 0.94; sensitivity = 0.88, AUC-ROC = 0.94), with the distribution of the mallard as, by far, the most important predictor of HPAI outbreak risk (Figure S1).

**Figure 3:**
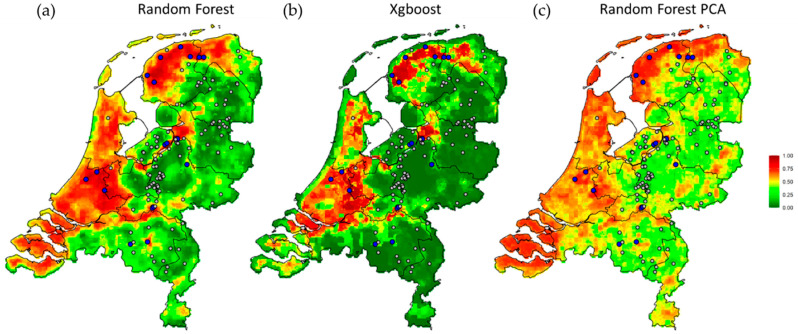
Mean farm outbreak probability of highly pathogenic avian influenza (HPAI) across all 1 km × 1 km grid-cells in the Netherlands for the three different algorithms; (**a**) the final leave-one-group-out random forest model (Random Forest); (**b**) the gradient boosted decision trees (Xgboost); and (**c**) the random forest on a PCA transformed feature set (Random Forest PCA). The prediction of HPAI risk ranges between 0 (low, dark green) and 1 (high, dark red). Locations of poultry farms with HPAI outbreaks (i.e., cases, blue) and control farms (grey) are shown.

The predicted spatial pattern of HPAI outbreak risk across the Netherlands (Figure 3) shows generally a high risk in the western and northern part of the Netherlands, as well as around the larger rivers in the center of the country (e.g., the Rhine, Waal, Ijssel and Meuse rivers). The eastern and southern parts of the Netherlands generally have a lower risk of HPAI outbreak. A negative correlation (−53.9%) between the standard deviation of the predicted infection probabilities (on a logit scale) and the average predicted farm outbreak probability was found, indicating that variation in the areas with high predicted HPAI risk was lower than in the areas where the infection risk was predicted to be low. 

The correspondence between the predicted risk maps (Figure 3) produced by different algorithms (the two random forests algorithms, with and without PCA-transformed feature set and the boosted decision trees with monotonicity constraints) indicates that the patterns captured in the risk maps are robust. Namely, removing the possible confounding effects of multicollinearity between predictors or enforcing positive monotonicity in the association between bird densities and HPAI outbreak risk did not lead to major changes in the predicted risk maps (correlation between random forest and gradient boosted decision trees: 84.3%; correlation between a random forest with all multiscale predictors and after PCA transformation: 79.5%).

## 3. Discussion

We showed that a model using wild bird species’ densities can accurately predict HPAI risk areas for poultry farms in the Netherlands. Seventeen waterbird and two raptor species were most strongly associated with the HPAI outbreak risks. 

The mallard and mute swan had the highest feature importance, and have both been found infected with HPAIVs, as described in several studies [4,5,9,21,26]. Other species selected by the model, e.g., Eurasian wigeon, peregrine falcon (*Falco peregrinus*) and great black-backed gulls (*Larus marinus*), were found to have high mortality rates during the H5N8 epidemic in 2016 [6], and wild birds of the family of Anatidae and of the order of Charadriiformes are often affected and may also play a role as important reservoir species for HPAIVs [27]. All bird species of which the densities contributed most to the prediction of HPAI outbreak risk for poultry farms were also among the confirmed HPAI wild bird cases in the EU between 2020–2022 [15,16], with the mute swan being one of the most frequently reported species in 2020–2021 [15] and 2021–2022 [16]. In addition to the mute swan, the top three affected species included the barnacle goose (*Branta leucopis*) and the graylag goose (*Anser anser*) [15,16]. These were not among the species mostly associated with HPAI outbreak risk for farms, suggesting that the species with the highest feature importance are not always similar to the wild bird species with the highest mortality rates from HPAIV infection (Table S1).

Our model outputs indicate which wild bird species’ densities were associated with the risk of infection for poultry farms, based on prior cases and a detailed wild bird count dataset covering the whole of the Netherlands, and was not based on reported numbers of infected or dead wild birds. The wild bird species indicated by the model may be relevant for HPAIV transmission, and, therefore, it may be important to consider these species as well when taking preventive measures around farms and for future studies. However, we have to be careful when drawing conclusions on the exact roles of specific wild bird species in the epidemiological processes at the wild bird/domestic bird interface, and on that of other bird species not included in this study. HPAIV infections among populations of wild bird species depend on a complex multi-species system, influenced by ecosystem properties, bird species diversity and community structure, the specific circulating HPAIV strain(s), and the clinical impact it has among the different hosts species [27,28,29]. This study does not indicate how many birds of the identified wild bird species were infected, or to what extent their presence contributed to disseminating the virus in the farms’ surroundings. For example, not all of the birds that were found often during passive surveillance between 2020–2022, such as the barnacle goose (*Branta leucopis*) and graylag goose (*Anser anser*) [15,16], were among the birds with the highest feature importance in the model. These species may have made a limited additional contribution to the model because of collinearity with other species, although such effects were limited by constraining the hyperparameters in the random forest model. Another explanation could be that birds that die quickly from HPAIV infection have a more limited role in disseminating the virus in the environment. In addition, it can suggest that the dynamics of species contributing most to HPAI outbreak risks may change over time, making it important to keep updating such models frequently with the newest data. Furthermore, the species selected by the model can include both migratory bird species (e.g., Eurasian wigeon), which likely play a role in long distance dispersal, and species that are less migratory (e.g., mute swan and mallard), which could act as local amplifiers, or bridge species [29,30,31]. The relative roles of different migratory, sedentary and synanthropic bird species in HPAIV’s introduction into poultry farms therefore remains to be elucidated.

The predicted higher HPAIV infection risk in the western and northern part of the Netherlands, and around larger rivers, is in the lowest parts of the Netherlands with soils containing more clay and peat, i.e., characteristic habitats for the more water-dependent bird species. In contrast, the higher and dryer eastern and southern parts of the Netherlands with generally sandy soils, have a lower risk of HPAI outbreak. Nevertheless, in contrast to previous studies [17,18,19,32,33], only one land cover variable, i.e., cover by agriculture, was selected in the final model, indicating that densities of wild bird species were better predictors of the HPAIV infection risk in our study. Previous studies mostly tried to explain the variation in the occurrence of HPAIV introductions with environmental variables, such as distance to waterways and vegetation index [18,19,33]. Others also included surveillance or telemetry data to track the movements of a selection of wild bird species in a certain time period [17,29,32,34,35,36], but most studies did not include detailed count data on wild bird species distribution to analyze HPAIV introductions on poultry farms. Environmental variables can be considered as a proxy for habitat suitability for wild birds, and were, in our study, less suitable predictors than the densities of the actual bird species. This does not mean that land cover data could not be of great value. Such data have proven valuable in many previous spatiotemporal analyses. For example, it has been shown in many studies that the presence of wetlands is vital as they are an important habitat for many waterfowl species [32,33,35]. In the current study, the original land cover classes were aggregated into five major classes, which decreased the resolution of land cover classification in the analyses, and may have reduced sensitivity of these variables for the prediction of farm outbreak probability. Therefore, especially in regions where detailed quantitative wild bird density data are not available, land cover data, and potentially also other ecological or climatic, bird habitat-related features that can be more easily gathered across large areas, could still be a suitable proxy for predicting HPAI risk.

Besides the spatial distribution of wild birds, seasonality and the arrival of migratory birds also play a role in the prediction of HPAI outbreak risk [20,30,36,37,38]. For example, Velkers et al. (2020) found that the timing of peak densities of Anatidae species observed around Dutch farms coincided with the timing of outbreaks [20]. In addition, reservoir host movement and behavioral states (e.g., resting, foraging, long-distance migration) can affect spatiotemporal overlap with poultry facilities and hence probabilities of HPAIV spillover, as demonstrated by the telemetry data of the blue-winged teal (*Spatula discors*) in the United States [36]. Hence, the spatiotemporal relationships between outbreaks on poultry farms and HPAI wild bird detections represent complex dynamics. In the current study, we only used long-term averages of bird count data, collected at set moments each winter between 2012/2013–2014/2015. Our model could be further improved by including temporal variation in local differences in densities of bird species, and by adding new HPAI cases on poultry farms, provided that these data are available, to further train and validate the model. 

The scale dependency of the different predictors was incorporated in all fitted algorithms. This is in line with ecological studies assessing the influence of environmental context, varied over different scales, on the analysis and prediction of habitat selection [39]. This is important, as local dispersal patterns and ecology of bird species differ. Diving ducks, e.g., tufted ducks, are mainly found on large open waterbodies, often at considerable distance from farms, and have relatively few movements over land between foraging and roosting sites. In contrast, Eurasian wigeons and mallards forage on grass- and agricultural lands, and are found more closely to farms [6,20], which is in line with our results. 

We realize that our dataset was limited, with 26 confirmed HPAI cases on poultry farms. Although some farms had multiple outbreaks over the years, these were all new introductions, and, thus, independent of one another. Furthermore, we tuned the random forest analysis in such a way as to minimize the risk of overfitting and used a cross-validation approach for the random forest, testing its robustness despite the limited dataset. Moreover, the potential problem of multicollinear predictors was successfully addressed by using the orthogonally transformed predictor-set through a PCA, and the gradient boosted decision tree algorithm successfully constrained the model to only monotonously increasing associations between bird densities and outbreak risk. The results of the latter two algorithms supported the random forest analysis, and the boosted decision tree even slightly outperformed the random forest analyses (Table 1), indicating that the spatial differences in mallard densities was the most important predictor. The mallard is among the most studied species with regard to LPAIV and HPAIV infection prevalence, proximity around farms and outdoor ranges [22] and movement patterns in relation to wetland and agricultural fields, as previously reviewed [40]. Combined with the current study, such knowledge can contribute to improving risk predictions and taking measures to reduce the risk of AIV introduction on poultry farms.

In conclusion, we show that spatial variation in HPAI outbreak risk in the Netherlands was accurately predicted based on wild bird density data, rather than on only land cover variables. The spatial distributions of several waterbird species were important contributors to model the HPAI outbreak risk. New HPAI outbreaks can be used to validate and improve the risk map, but already in its current form, areas classified as high risk for HPAIV introduction on poultry farms should be considered as important targets for surveillance, preventive measures against HPAIV introduction, and may assist in decision making on the locations for new poultry farms. The described modelling approach allows for inclusion of the best predictors based on the available data, which may include land cover variables in addition to bird data, depending on the local situation. Identification of high risk areas for development of country- or region-specific control programs would be a proactive strategy to combat the global threat of these recurring HPAI outbreaks. 

## 4. Materials and Methods

### 4.1. Study Design

#### 4.1.1. Case Farms

A case control study was performed retrospectively, using all 26 diagnosed HPAIV H5N8, H5N6 and H5N1 infections on poultry farms in the Netherlands between the autumn–winter periods of 2014/2015, 2016/2017, 2017/2018 and 2020/2021. Ten outbreaks were on layer farms, eight on Pekin (meat production) duck farms, five on broiler breeder farms, two on broiler farms and one on a turkey (meat production) farm. Some farms were affected repeatedly in different years (Table 2). These multiple outbreaks occurred in different years and were all primary introductions, i.e., assumed to be as a result of wild bird to poultry transmission and not secondary between-farm spread, based on the genetic analyses of the viruses and epidemiological investigations [4,5,20,21,26]. 

#### 4.1.2. Control Farms

All farms that were not confirmed HPAI cases can be considered free of HPAIV infection, as a strict reporting system obligates farmers to notify any signs of HPAIV infection, and regular serological testing is executed on all poultry farms in the Netherlands to confirm absence of subclinical infection [4,5,20,21,26]. We randomly selected four unique uninfected poultry farms from the Netherlands Food and Consumer Product Safety Authority (NVWA) database for each HPAI case farm in every year. Control farms were selected based on similar poultry type to the infected case farm (i.e., layer, Pekin duck, broiler breeder, broiler or turkey farm), registration as an active poultry farm in the same year as the outbreak year in the case farm and located within a 100 km radius of the case farm. Farms that had been a case in any of the years were excluded for control selection and controls could only be selected for one case farm.

### 4.2. Selection of Wild Bird Density Data

We reviewed the literature for wild bird associations with HPAIV infection from the Netherlands, but also from other countries [1,5,6,41,42,43], to compile a list with species that had a known association with HPAIV infection (Table S1). Bird species taxonomically close to the species that had been associated with HPAIV infection were also included. Bird species that were rare (<500 individuals in the winter counts across the Netherlands), had a small geographical range (e.g., only present in the Wadden Sea) or only present in summer months, were excluded to prevent spurious negative associations. 

### 4.3. Bird Count Data

Bird count data collected by Sovon, the Dutch Centre for Field Ornithology (Nijmegen, the Netherlands), and published in the *Bird atlas for the Netherlands* [44], were used for the analyses. The *Bird atlas for the Netherlands* was compiled through, among other things, structured bird counts across the whole of the Netherlands by (largely voluntary) observers in three winter seasons from December–February 2012/2013–2014/2015. We only used winter bird density data, as HPAI outbreaks only occurred between November and March. In short, for organizing the field work and processing the data the whole of the Netherlands was divided into 5 km × 5 km squares. Each square was assigned to an observer who performed bird counts in pre-defined months. Sovon used the obtained data to construct maps with an estimated numbers of wild birds per species per square (Appendix A). For 54 of the 58 bird species of interest the estimates per square were available. For four bird species (i.e., mallard, Eurasian magpie, carrion crow and western jackdaw) the maps with estimates per square did not pass the internal review process, and we therefore transformed the relative abundance (density) maps with 1 km × 1 km resolution, into maps with the estimated number of the particular species per 5 km × 5 km square. 

### 4.4. Land Cover Data

Land cover data (LGN7) were available as a raster layer with 25 m resolution, from which we computed the fractional cover per 5 km × 5 km square, which aligned with the bird count data. We selected land cover classes based on relevance for the distribution of high risk HPAI bird species and HPAI risk on poultry farms [17,19]. These land cover classes were aggregated into five major classes (agriculture, freshwater systems, grasslands, swamps and peat area and saltwater systems) to reduce the number of classes to be used for further analyses (Table S2). 

### 4.5. Including Spatial Context 

All downstream analyses were performed in R (version 4.1.0) [45]. We resampled all 5 km × 5 km squares to 1 km × 1 km resolution using nearest-neighbor allocation, so that all grid cells within each 5 km × 5 km square contained the same value. To include the influence of landscape context (i.e., bird density and land cover predictors) beyond the properties of the grid cell, we applied isotropic bivariate Gaussian smoothers to the grid layers, with bandwidths of 2.5, 5 and 10 km [46,47]. The unsmoothed raster layers contained essentially no information on the environmental context beyond the 5 km × 5 km square, and thus only contained site-specific information.

### 4.6. Cross-Scale Analyses and Dimension Reduction

We thus had a large number of features (252: 5 land cover classes; densities of 58 bird species, each at four spatial scales, including the raw unsmoothed rasters), compared to a limited number of cases (*n* = 26). To reduce the number of potential predictors, we first performed a series of univariate conditional logistic regressions (CLR, using the survival package, [48]), with cases and matching controls stratified by case farm identifier. All features were standardized to zero-mean and unit-variance prior to analyses. We then applied feature selection by omitting features from further analyses when either (a) there was insufficient variation in the feature values over the case-control points, or (b) when the CLR indicated that feature values were negatively associated with HPAI outbreak risk. This latter filtering step only applied to the bird density features, in order to avoid spurious and uninterpretable negative association. All land cover classes and 51 bird species passed this step of filtering (Table S1). 

Then, in order to achieve further dimension reduction, we merged the rasters measured at different scales within a bird species or land cover class into a single raster layer by computing the CLR slope-weighted sum of the individual scales. In addition, these scale-aggregated predictors were standardized to zero-mean and unit-variance. Moreover, CLR analyses showed that these predictors were all positively associated with HPAI outbreak risk. 

### 4.7. Model Training and Evaluation 

We performed predictive analyses using a leave-one-group-out cross validation approach, where a group consists of a unique poultry farm on which HPAI occurred (i.e., the cases) together with its matched controls. We used the following machine learning algorithms and data preparation steps. We trained a random forest classifier (using the ranger package, [49]) on all multiscale predictors. Random forests generally deal well with large numbers of input variables, and when tuned well they can be robust against overfitting [50]. We tuned the following hyperparameters in a grid search: number of variables per tree (tuning range: 2–9; tuned hyperparameter value: 8); minimum node size (1–4; 2); and maximum tree depth (2–9; 8), all given a fixed forest size of 100,000 trees. In order to put more emphasis on high recall (i.e., sensitivity or true positive rate) at the expense of having lower precision, we evaluated predictive performance using a weighted F1 score, where recall was weighed four times higher than precision. We then trained the algorithm using the optimal hyperparameters, and quantified feature importance using the permutation variable importance approach [51]. Feature importance values were scaled by dividing them by the mean importance value, so that the average feature importance value was 1 (Table S1). Predictive performance was assessed using the area under the curve (AUC) of the receiver operating characteristic (ROC) curve (a threshold independent metric that captures the trade-off between sensitivity and 1-specificity), as well as threshold-dependent metrics such as accuracy and sensitivity (recall). A prediction of the HPAI risk across the Netherlands was made by averaging the cross-validated predicted HPAI probability surfaces.

Furthermore, to account for the potential problem of the correlated feature set, we trained another random forest classifier following the procedure as outlined above, yet with a principal components analysis (PCA) as an intermediate step. The PCA orthogonally transformed the predictor feature set into a new feature set with statistically independent principal components, where we kept all dimensions, and thus kept all variation present in the original predictors. 

Moreover, to prevent uninterpretable associations between bird densities and HPAI outbreak risk, we also trained a gradient boosted decision tree algorithm (XGBoost package), where we only allowed monotonously increasing associations between bird densities and HPAI outbreak risk. By comparing the predictions of these three different algorithms, we assessed the robustness of the random forest classifier on the original multiscale predictors by comparing its predictions to the ones generated by the other two algorithms.

## Figures and Tables

**Table 1:** Classification metrics for predicted highly pathogenic avian influenza risks (Figure 3) for the different algorithms: random forest (RF), gradient boosted decision trees (GBT), and random forest on a PCA transformed feature set (RF-PCA). All classification metrics except for the AUC- ROC are for a cut-off threshold that maximizes the weighted F1 score, which was 0.278 for this model. AUC-ROC is the area under the receiver operating characteristic curve, a threshold-independent performance measure.

Algorithm	Accuracy	Recall/Sensitivity	AUC-ROC
RF	0.86	0.88	0.92
GBT	0.94	0.88	0.94
RF-PCA	0.87	0.81	0.88

**Table 2:** Overview of highly pathogenic avian influenza (HPAI) cases in the Netherlands on individual farms (ID 1 to 21) with confirmed HPAIV infection between 2014–2021. Poultry type indicates the type of farm that was affected. On poultry farms 1, 4 and 6 multiple HPAI outbreaks were diagnosed between 2014–2021.

Case-ID	Poultry Type	2014-H5N8	2016-H5N8	2017-H5N6	2018-H5N6	2020-H5N8 ^1^
1	Layer	x	x			x
2	Layer	x				
3	Layer	x				
4	Pekin Duck	x	x		x	
5	Broiler Breeder	x				
6	Pekin Duck		x	x		
7	Pekin Duck		x			
8	Pekin Duck		x			
9	Layer		x			
10	Layer		x			
11	Broiler Breeder		x			
12	Broiler Breeder				x	
13	Broiler Breeder					x ^1^
14	Layer					x
15	Layer					x
16	Pekin Duck					x
17	Broiler					x
18	Broiler					x
19	Broiler Breeder					x
20	Turkey					x
21	Layer					x

^1^ The broiler breeder case in 2020–2021 was diagnosed with HPAIV H5N1. All other HPAI farms between October 2020 and February 2021 were confirmed with HPAIV H5N8.

## Data Availability

Not applicable.

## Supplementary Materials

The following supporting information can be downloaded at: https://www.mdpi.com/article/10.3390/pathogens11050549/s1, Figure S1: Feature importance of 20 most important scale-aggregated predictors according to the gradient boosted tree with positive monotonicity constraints; Table S1: Overview of candidate variables in order of (Rank) decreasing feature importance, including a selection of high risk bird species for HPAIV infection and land cover variables; Table S2: Aggregation of land cover classes to five major classes used in the random forest algorithms.

## Appendix A. Bird Count Data Collection and Analyses

Dutch bird count data collected by Sovon, the Dutch Centre for Field Ornithology (Nijmegen, The Netherlands), and published in the *Bird atlas for The Netherlands* [44] were used for the analyses. The *Bird atlas for The Netherlands* was compiled through a citizen science approach of structured bird counts across the whole of The Netherlands by (largely voluntary) observers in three winter seasons from December–February 2012/2013–2014/2015. We only used winter bird density data, as HPAI outbreaks only occurred between November and March. For organizing the field work and processing the data the whole of The Netherlands was divided in 5 km × 5 km squares (1769 squares in total, including the open water areas of lake IJsselmeer, the Wadden Sea and the large river deltas). Each square was assigned to an observer who performed bird counts in pre-defined spring/summer and winter months. Each observer was instructed to perform a minimum of three bird counts in the assigned square between December and February, visiting all main habitats within the square. As a final result after counts were finished, the observer made an estimate of the number of individuals per bird species present in the square in the winter according to the following classes: 1–3; 4–10; 11–25; 26–50; 51–100; 101–250; 251–500; 501–1000; >1000. Sovon used the obtained data to construct maps with numbers per species per square based on the estimated numbers per square. An internal reviewing process checked the constructed maps for each bird species. Quality checks entailed checking if the pattern of distribution of a bird species over The Netherlands was accurate compared to what is known from the distribution of the species from other projects lead by Sovon.
