# Supplementary material for: Wild Bird Densities and Landscape Variables Predict Spatial Patterns in HPAI Outbreak Risk across The Netherlands
Source: Pathogens. 2022 May 6;11(5):549. doi: 10.3390/pathogens11050549 (PMC9143584; doi:10.3390/pathogens11050549)
Supplement: Supplementary file 1 [file pathogens-11-00549-s001.zip › FigS1_Schreuder_SpatialriskHPAI_220506.pdf]

Wild Bird Densities and Landscape Variables Predict Spatial Patterns in HPAI Outbreak Risk across The Netherlands

Janneke Schreuder, Henrik J. de Knegt, Francisca C. Velkers, Armin R. W. Elbers, Julia Stahl, Roy Slaterus, J. Arjan Stegeman and Willem F. de Boer

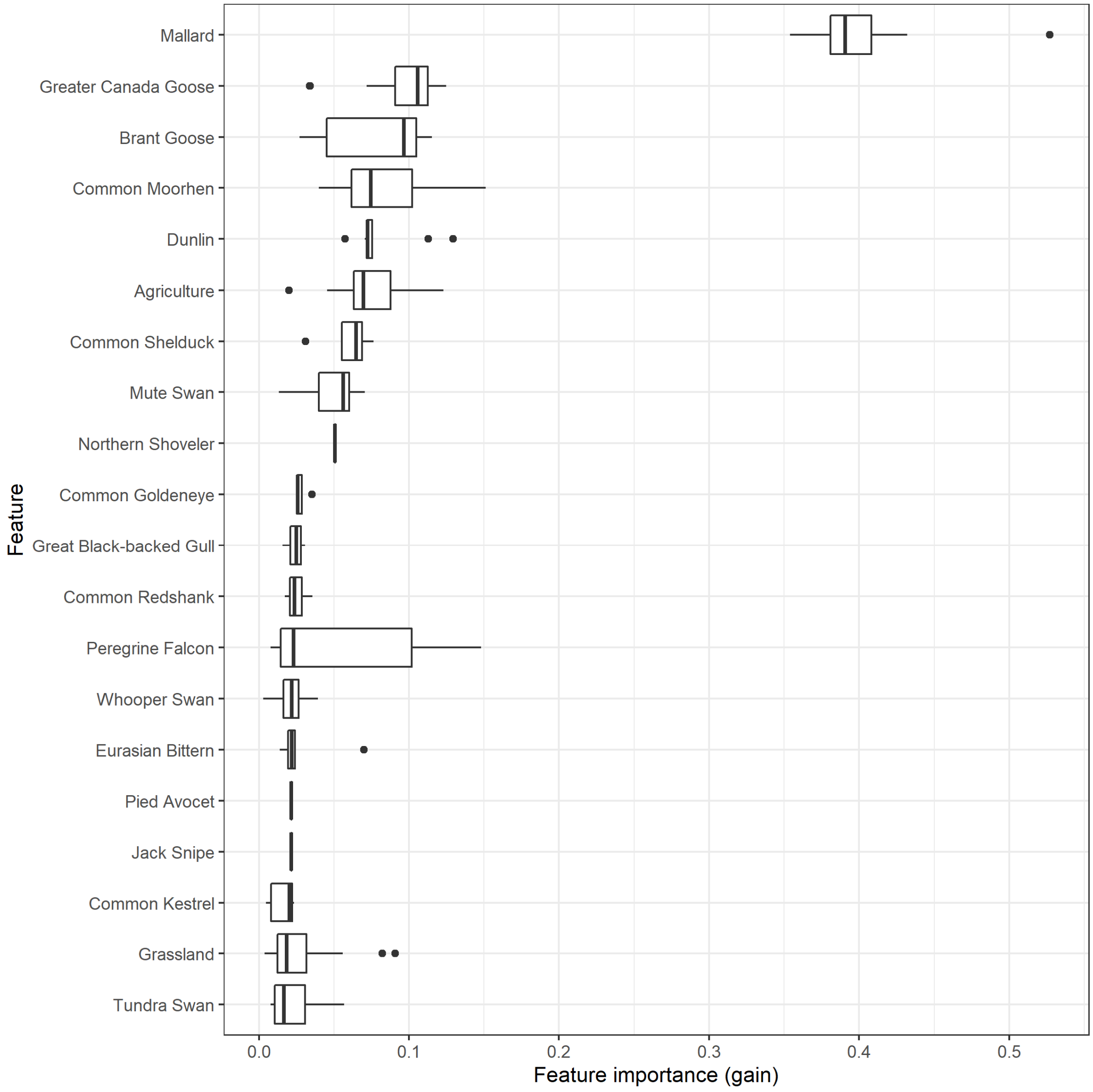

Figure S1. Feature importance of 20 most important scale-aggregated predictors according to the gradient boosted tree with positive monotonicity constraints.
